# Precision in Tear Fluid Biomarker Discovery: Quantitative Proteomic Profiling of Small-Volume, Individual Samples Using Capillary Tube Collection

**DOI:** 10.3390/biomedicines13020386

**Published:** 2025-02-06

**Authors:** Kyla Frenia, Yunxiang Fu, Maria A. Beatty, Kathleen C. Garwood, Jeremy Kimmel, Veena Raiji, Dipanjan Pan, David Bartlett, Leanne T. Labriola, Kunhong Xiao

**Affiliations:** 1 Department of Bioengineering, Swanson School of Engineering, University of Pittsburgh, Pittsburgh, PA 15260, USA; frenia@innsightech.com (K.F.); jdk33@pitt.edu (J.K.); 2 Allegheny Health Network Cancer Institute, Pittsburgh, PA 15212, USA; fuyunxia@grinnell.edu (Y.F.); david.bartlett@ahn.org (D.B.); 3 Center for Proteomics & Artificial Intelligence, Allegheny Health Network Cancer Institute, Pittsburgh, PA 15202, USA; 4 Center for Clinical Mass Spectrometry, Allegheny Health Network Cancer Institute, Pittsburgh, PA 15202, USA; 5 Retina Service, Sewickley Eye Group, Sewickley, PA 15143, USA; maria@pghtrials.com; 6 Department of Decision and System Sciences, Saint Joseph’s University, Philadelphia, PA 19131, USA; kcampbel@sju.edu; 7 Department of Ophthalmology, Rush University Medical Center, 1725 W. Harrison St., Suite 915, Chicago, IL 60612, USA; veena.raiji@gmail.com; 8 Huck Institutes of the Life Sciences, Department(s) of Biomedical Engineering, Nuclear Engineering, Materials Science and Engineering, The Pennsylvania State University, 101 Huck Life Sciences Building, University Park, PA 16802, USA; dipanjan@psu.edu; 9 Department of Biomedical Engineering, College of Engineering, Carnegie Mellon University, Pittsburgh, PA 15213, USA

**Keywords:** tear proteomics, tear biomarker, biomarker discovery, capillary collection, LC-MS/MS

## Abstract

**Background**: Tear fluid, rich in proteins, is a promising source of novel biomarkers for ocular and systemic health. Liquid chromatography-tandem mass spectrometry (LC-MS/MS) is the primary method for biomarker discovery. Still, factors such as limited sample volume, extracellular protein contamination, and reflex tearing can significantly impact results. Glass microcapillary tubes minimize these issues. Schirmer strips remain the most common collection method due to existing LC-MS/MS protocol optimization. **Methods**: In this study, we evaluated multiple digestion protocols for the shotgun quantitative LC-MS/MS analysis of small-volume tear fluid samples collected using glass capillary tubes. Protocol optimization was performed using pooled samples and then compared with the analysis of individual samples. **Results**: Using the optimized protocol, one μL samples were processed using a timsTOF Pro 2 mass spectrometer (Bruker) coupled online with an Evosep One liquid chromatography system (Evosep), leading to the identification of an average of 361 ± 63 proteins in pooled samples and 525 ± 123 proteins in individual small-volume tear fluid samples. **Conclusions**: This protocol highlights the practicality of using glass capillary tubes for comprehensive LC-MS/MS-based tear proteomics analysis, paving the way for detailed proteomics characterization of individual tear fluid samples rather than pooled samples. By shifting from pooled to individual samples, this approach greatly accelerates tear biomarker discovery, advancing precision and personalized medicine.

## 1. Introduction

Tear fluid is a complex biological matrix that comprises proteins, lipids, metabolites, and small molecules that support ocular health and function [1,2]. Basal tear fluid is continuously replenished, and this fluid helps to maintain the ocular surface by providing lubrication, essential nutrients, and immunological protection [3]. Basal tear proteins are primarily produced by the lacrimal and meibomian glands, with contributions from ocular surface epithelial cells and blood plasma filtrates [4,5]. This composition results in a highly diverse proteome, with thousands of proteins that exhibit distinct protein signatures in response to various ocular [6,7,8,9] and systemic diseases [10]. Tear fluid is a highly attractive target for point-of-care diagnostic applications because the collection of tear fluid can be performed non-invasively and requires minimal training [11].

However, to use tear fluid as a reliable reservoir for diagnostic testing, strategies for improving the reproducibility and consistency of fluid collection need to be identified. Tear fluid is dynamic, and its composition is influenced by various factors, such as biological elements like age, sex, and genetics, as well as environmental influences including exposure to different conditions and lifestyle choices. Temporal factors, like the time of day or year, also play a role [12]. While these sources of variation are common in many biological samples, tear fluid is particularly prone to additional variability due to sample collection and processing [11]. Challenges such as low sample volume, reflex tearing, and the instability of the tear layer in individuals with ocular or systemic diseases further complicate tear fluid analysis [2,4,13].

The most common method of tear collection used in proteomic studies is Schirmer strips. Schirmer strips are small, standardized filter paper strips placed on the lower eyelid to collect tear fluid. They are commonly used to assess tear production by measuring the wetted length after a set period, and the absorbed fluid can also be analyzed for biomarkers. They are widely used for tear fluid collection because they are simple to administer, are easy to obtain, and can absorb a significant amount of tear fluid [14,15]. However, there are several drawbacks to the use of Schirmer strips for proteomic analysis. First, the contact of the Schirmer strip with the eyelid during collection induces reflex tearing [16,17]. Reflex tears are produced by the lacrimal gland in response to mechanical stimulation or eye irritation. This reflex of tears can dilute or alter the composition of the tear fluid sample [13,18]. Additionally, contact with the lid increases the risk of introducing cellular contamination [19]. Collection with Schirmer strips can also be difficult to standardize, as variations in tear flow and technique can impact results. Sample collection is typically controlled by timing the duration the strip is left in place or setting a preferred wetting length, but these methods may introduce variability, particularly in patients with very low or high tear production.

The second most common method of tear collection is glass microcapillary tubes. Glass capillary tubes are less likely to cause cellular contamination—a critical factor in proteomic analysis, where cellular debris can interfere with results. Additionally, glass capillary tubes are generally less invasive and more comfortable for patients compared to Schirmer strips, making them a better option in clinical settings where patient comfort is a priority [11]. Despite these advantages, the methods for processing and analyzing tear fluid samples collected with glass capillary tubes remain underdeveloped compared to those used for Schirmer strips [20]. This is because the analysis of capillary samples presents several unique challenges for proteomic discovery. The small volumes typically obtained through capillary tubes, often less than one microliter, pose challenges for downstream processing steps such as protein quantification, desalting, and enzymatic digestion. These limitations can result in protein loss, increased variability, and reduced sensitivity in liquid chromatography–tandem mass spectrometry (LC-MS/MS) analysis [11,12,15].

The absence of standardized protocols for processing tear fluid collected by capillary tubes exacerbates these issues, as sample preparation methods optimized for larger fluid volumes, like those obtained with Schirmer strips, may not be directly applicable to the smaller samples obtained from capillary tubes [21]. Therefore, there is a critical need for the development of optimized and standardized protocols specifically tailored to the unique properties of tear fluid collected by capillary tubes [12]. Addressing these challenges is essential for realizing the full potential of tear fluid proteomics in the discovery and validation of novel biomarkers for ocular and systemic diseases. To address this, our study developed an optimized digestion protocol for proteomic analysis of tear fluid collected with glass capillary tubes, aiming to enhance biomarker discovery by improving protein identification and reducing technical variation.

## 2. Materials and Methods

### 2.1. Sample Collection

**Figure 1:**
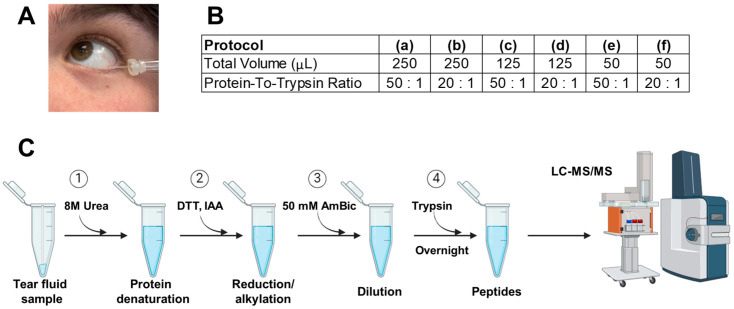
Collection and processing protocols for tear fluid samples. (**A**) Collection of tear fluid with glass capillary tubes is non-invasive and requires minimal training. An amount of 0.5 µL glass capillary tubes was used for all collections. (**B**) The protein-to-trypsin ratio and total digest volume for the six protocols evaluated in this study were as follows: (a) (50:1, 250 μL), (b) (20:1, 250 μL), (c) (50:1, 125 μL), (d) (20:1, 125 μL), (e) (50:1, 50 μL), and (f) (20:1, 50 μL). Five runs were performed for each protocol, with each run consisting of processing a single 1 μL aliquot of the pooled sample. (**C**) Experimental procedures for protein denaturation, reduction, alkylation, and trypsin digestion. Protocols tested differed in steps 3 and 4 as shown. In step 3, the total digestion volume was varied. In step 4, the protein: trypsin ratio was modified. The glass capillary with the 0.5 μL tear sample was transferred into a Protein LoBind microcentrifuge tube, where the entire procedure was carried out to minimize sample loss and reduce errors associated with handling small volumes. Low-speed centrifugation was used to extract the tear sample from the capillary tubes without breaking.

Tear fluid samples were collected using 0.5 μL glass microcapillary tubes (Microcaps^®^ Disposable Micropipettes, Drummond, PA, USA) (Figure 1A) from human volunteers. A maximum of two capillary tubes were collected from each subject. Sample collection was conducted in accordance with the ethical principles outlined in the Declaration of Helsinki, with approval from the relevant institutional review board (IRB number Pro00072069) and informed consent obtained from all participants. The microcapillary tube was placed into the lower conjunctival sac of the eye. This area is located between the lower eyelid and the eye’s surface. The insertion was performed gently and without contacting the lid to minimize reflex tearing and sample contamination by intracellular proteins, as well as to minimize discomfort. No anesthetic was applied before collection. Sampling took place during typical clinic hours, between 9:30 a.m. and 5:00 p.m. Subject samples were not contact lens wearers and did not receive or self-administer any ophthalmic drops within 30 min of collection. Samples were stored at −80 °C until processing.

Tear fluid samples were extracted from the glass capillary tubes through low-speed centrifugation. Capillary tubes were placed in an Eppendorf tube and centrifuged at 2000× *g* 2 min. This method extracted the tear fluid without flushing, which can cause sample dilution.

For method optimization experiments, samples were pooled to create a single uniform sample. A total of seventy-nine 0.5 μL capillary samples were collected from thirty-five individuals (mean age = 63 ± 19 years; 19 males, 16 females) and combined to create the pooled sample. Subjects included in the pooled samples included healthy subjects, as well as subjects with a history of ocular disease (Table 1). The pooled sample was then divided into 1 μL aliquots for processing. This pooled sample served as a standardized material for assessing the number of proteins identified and the consistency of results across the six protocols, removing the effects of typical sources of variability, including biological, environmental, and temporal factors.

Additional samples were collected from fourteen healthy control subjects for individual analysis (mean age = 53 ± 23 years; 2 males, 12 females) (Table 1); a 1 μL sample was collected from each of the 14 subjects and processed. The ages of the subjects included in the pooled sample and individual sample sets were not statistically different (Welch’s Test, *p* = 0.152).

### 2.2. In-Solution Protein Digestion

Using the pooled and aliquoted tear samples (1 μL each) prepared above, we designed and systematically evaluated six tear proteomics protocols. These protocols were specifically developed to test combinations of varying digestion volumes and protein-to-trypsin ratios (Figure 1B). In addition, we established a streamlined in-solution digestion method tailored to the unique characteristics of tear fluid samples (Figure 1C).

The protein-to-trypsin ratio and total digest volume were varied across six protocols: (a) (50:1, 250 μL), (b) (20:1, 250 μL), (c) (50:1, 125 μL), (d) (20:1, 125 μL), (e) (50:1, 50 μL), and (f) (20:1, 50 μL) (Figure 1B,C). Previous experiments optimized column loading mass, gradient length, and urea volume. Both digest volume and protein-to-trypsin ratio are particularly important to optimize for tear fluid samples due to their low protein concentration and small sample volume, which require precise enzyme conditions to ensure efficient, consistent digestion without over- or under-digestion. The conditions tested were selected for testing in an optimization experiment to cover a range of digestion conditions that balance enzyme activity and protein breakdown efficiency. The higher protein-to-trypsin ratios (50:1) and larger digest volumes (250 μL) ensure sufficient trypsin for digesting low-abundance proteins from the small 1 μL tear fluid samples, while the lower ratios (20:1) and smaller volumes (50 μL) test the limits of trypsin usage and concentrate the reaction for more efficient digestion. Testing these varied conditions allows for fine-tuning the digestion process to achieve optimal peptide generation while avoiding over-digestion or excess trypsin consumption.

Protein samples were prepared and digested as follows: 10 μL of 8 M urea in 50 mM ammonium bicarbonate (pH 8) (Millipore Sigma, Burlington, MA, USA, Catalog No. 09830-500G) was added to each sample tube. The mixture was vortexed and then centrifuged at 3000× *g* for 30 s. The 8 M urea solution was prepared by dissolving 480 mg of urea in 50 mM ammonium bicarbonate and adjusting the final volume to 1 mL. Protein concentration was determined using a Nanodrop spectrophotometer at 280 nm. To break disulfide bonds, 1 μL of 500 mM dithiothreitol (DTT) (Millipore Sigma, Burlington, MA, USA, Catalog No. 3860-5GM) was added to the sample, followed by vortexing, centrifugation at 3000× *g* for 30 s, and incubation at 56 °C for 30 min. Alkylation was achieved by adding 1 μL of 750 mM iodoacetamide (IAA) (Millipore Sigma, Burlington, MA, USA, Catalog No. I6125-5G), vortexing, centrifuging at 3000× *g* for 30 s, and incubating in the dark at room temperature for 45 min. The 750mM IAA solution was prepared by dissolving 13.9 mg of IAA in 100 μL of HPLC-grade water. Alkylation was subsequently quenched with 1 μL of 500 mM DTT, followed by vertexing, centrifugation, and incubation at room temperature for an additional 30 min. The sample was then diluted with 50 mM ammonium bicarbonate to reach the total digest volume specified by each protocol, vortexed, and centrifuged. For digestion, Trypsin/Lys-C mix (Mass Spec Grade, Promega, Madison, WI, USA, Catalog No. V5071) was added according to the protein–trypsin ratio specified by each protocol. A trypsin stock solution was prepared by dissolving 20 μg of trypsin in 100 μL of 50 mM ammonium bicarbonate to achieve a concentration of 0.2 μg/μL and stored at −30 °C. Samples were incubated with trypsin at 37 °C for 16 h. Digestion was stopped by adding 10% formic acid (LC-MS grade, ThermoFisher, Waltham, MA, USA, Catalog No. 28905) to adjust the pH to 3–4, as verified with pH strips, and the digested sample was stored at 4 °C.

### 2.3. LC-MS/MS

MS raw data were collected using a timsTOF Pro 2 mass spectrometer (Bruker, Billerica, MA, USA) online coupled with an Evosep One liquid chromatography system (Evosep, Odense, Denmark). A nano-LC setup with an 8 cm Evosep performance column (EV-1109) was used for peptide separation. The tear peptide samples were analyzed using a standard data-dependent acquisition method with parallel accumulation-serial fragmentation (DDA-PASEF) and the instrument operating in positive mode. A full scan was first acquired with a mass range of 100–1700 *m*/*z* and a TIMS 1/k0 range of 0.60–1.60 V·s/cm^2^. During a full 1.17 s cycle, 10 PASEF ramps were performed, with ramp and accumulation times both set to 100 ms. The following DDA-PASEF parameters were applied: precursor intensity threshold: 2.5 × 10^3^; charge state: +1 to +5; dynamic exclusion: 0.4 min; target intensity for fragmentation: 2 × 10^4^; isolation window (linear): 2 *m*/*z* at 700 *m*/*z* and 3 *m*/*z* at 800 *m*/*z*; collision energy (linear): 20 eV at 1/k0 of 0.60 V·s/cm^2^ and 59 eV at 1/k0 of 1.60 V·s/cm^2^.

### 2.4. Protein Identification and Analysis

All DDA raw datasets were analyzed using MaxQuant (version 2.1, Max-Planck-Institute of Biochemistry, Germany) and searched against the UniprotKB/Swiss-Prot reviewed human protein database (downloaded 30 March 2023). Peptide–spectrum matches (PSMs) were evaluated using the MaxQuant score method, which assesses the quality of the match between observed and theoretical spectra. Peptides were validated based on a minimum score of 20 for unmodified peptides and 40 for modified peptides, with an additional delta score cut-off of 6 applied for modified peptides. A False Discovery Rate (FDR) of 1% (0.01) was applied at both the peptide and protein levels to minimize false positives. Trypsin specificity was set to cleave peptide bonds at the carboxyl side of lysine (K) and arginine (R), except when either of these residues is followed by proline (P), allowing up to two missed cleavage sites. A maximum of two of the following dynamic modifications were allowed on each peptide: C [+57.021464], STY [+79.966331], and M [+15.994915]. Precursor and fragment mass tolerances were set to ±10 ppm and ±25 ppm, respectively. For protein identification, at least one associated peptide was identified within ±5 ppm of its theoretical *m*/*z* value.

### 2.5. Statistical Analysis

The Kruskal–Wallis test was applied to evaluate overall differences in the average protein and peptide counts across the six protocols. When significant differences were observed, the Mann–Whitney U test was used for pairwise comparisons to identify specific protocols with significant differences in the number of identified proteins and peptides. To assess run-to-run variance—with each run involving the processing of a single 1 μL aliquot of the pooled sample—we calculated the coefficient of variation (CV%) within each protocol. CV% was determined only for proteins that were identified in every run across all six protocols. The intra-protocol CV% for each protein was then compared using a paired *t*-test. A similar method for comparing variance was used to compare the variance of protein identified in the HC individual samples and the pooled sample runs for protocol (f). A *t*-test was used to compare the number of proteins identified in pooled runs for protocol f and the individual samples.

### 2.6. Selection of Optimal Protocol

**Figure 2:**
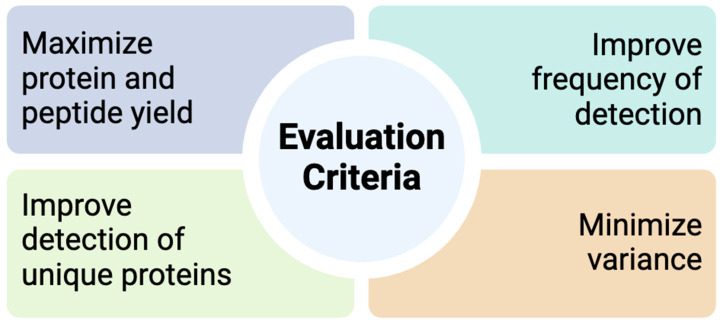
Evaluation criteria for method optimization and selection. The goals of method optimization are to maximize protein and peptide yield, improve the detection of unique proteins, improve the frequency of detection for proteins, and minimize variance.

To select the optimal protocol for proteomic analysis, a comparison was made using standardized evaluation criteria. These included (i) maximizing protein and peptide yield, (ii) improving the detection of unique proteins, (iii) achieving higher detection frequency across samples, and (iv) minimizing variability in results (Figure 2). The protocol demonstrating the best overall performance across these criteria was selected for its ability to provide sensitive, consistent, and reproducible proteomic data suitable for biomarker discovery and clinical applications.

## 3. Results

### 3.1. Protein and Peptide Yield

**Figure 3:**
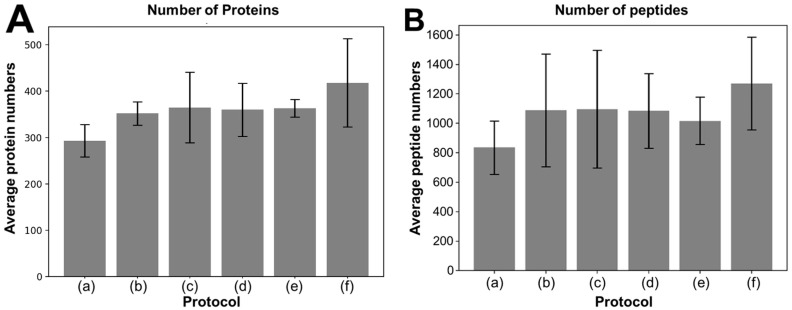
Comparison of identified protein and peptide numbers across six different digestion protocols (a)–(f). (**A**) Comparison of identified protein numbers across six protocols. Protocol (f) yielded the highest average number of unique proteins per run. (**B**) Com parison of identified peptide numbers across six protocols. Protocol (f) also identified the highest average number of peptides per run.

A total of 1022 proteins were identified in this study across 30 pooled and 14 individual samples (Supplemental Table S1). For method optimization, a total of 30 processing runs were completed, with five runs conducted for each of the six experimental protocols. These runs identified 741 proteins in total (Supplemental Table S2). On average, protocol (f) identified the greatest number of proteins, with 418 ± 95 proteins detected per run, while protocol (a) identified the fewest proteins, with 293 ± 35 proteins (Figure 3A and Table 1). Statistical analysis revealed that protocol (a) identified a significantly lower number of proteins compared to protocols (e) (*p* = 0.001) and (f) (*p* = 0.003). However, no statistically significant differences in the number of proteins identified were observed between any of the other protocols. In terms of peptide identification, protocol (f) again performed the best, detecting an average of 1270 ± 314 peptides per run, while protocol (a) detected the fewest, with 835 ± 181 peptides (Figure 3B and Table 2). However, no statistically significant difference in the number of peptides identified was observed between protocols (*p* = 0.38). Protocol (f) exhibited superior performance in maximizing protein and peptide yield compared to the other protocols tested.

### 3.2. Unique Protein Identification

**Figure 4:**
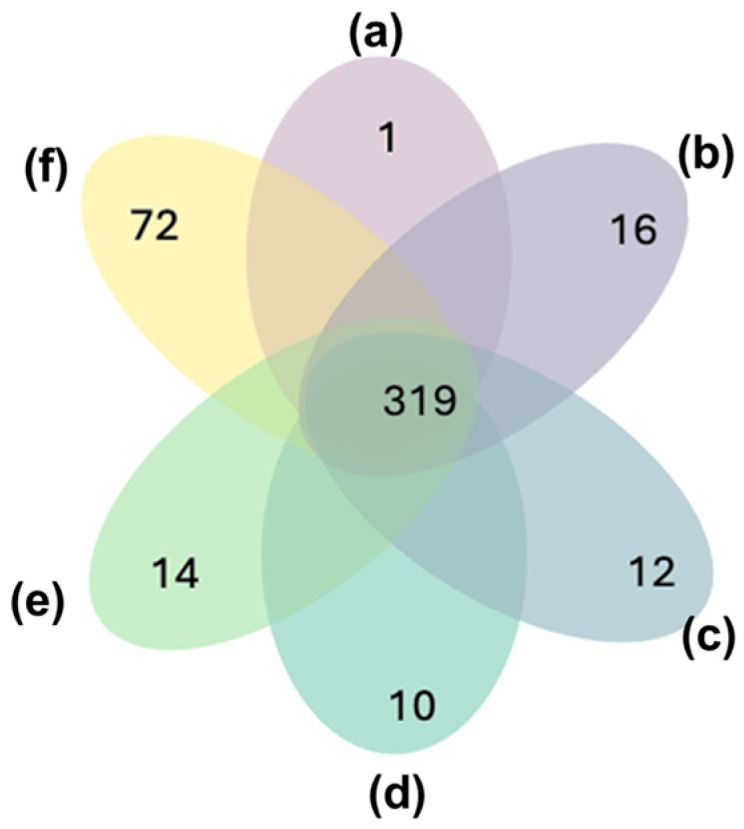
Comparison of unique proteins identified across six digestion protocols. A total of 319 proteins were consistently identified across all six protocols. Among the protocols, protocol (a) demonstrated the lowest ability to identify unique proteins, detecting only one protein not found in any other protocol. In contrast, protocol (f) showed the highest capability for identifying unique proteins, with 72 proteins exclusively detected by this protocol.

Of the proteins identified, 319 were consistently identified in at least one run per protocol, with 73 proteins reliably detected across every single run. Notably, protocol (f) emerged as the most effective in terms of the number of proteins identified, yielding 72 proteins exclusively detected using this protocol (Figure 4). This result highlights protocol (f)’s ability to maximize the proteomic coverage of tear samples, likely due to its optimized digestion volume and protein-to-trypsin ratio.

### 3.3. Frequency of Detection

**Figure 5:**
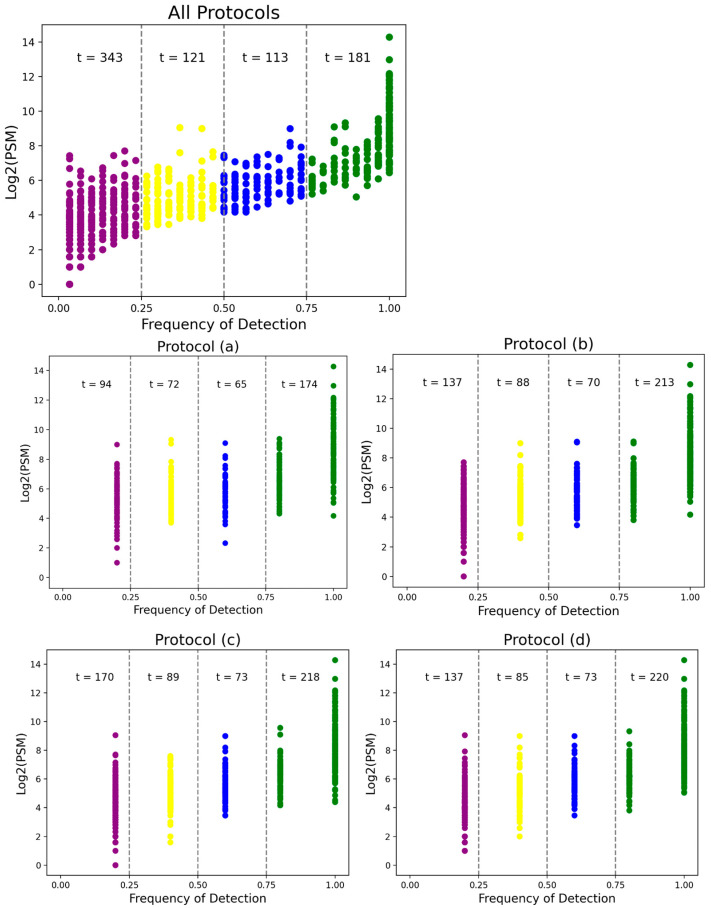

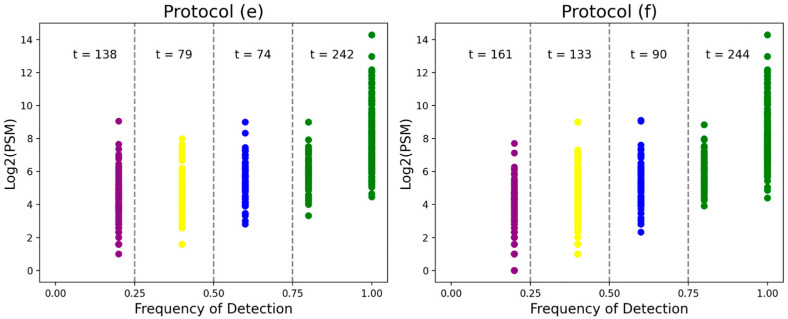
The frequency of proteins identified across processing runs plotted against abundance, as indicated by log2(PSM). Total protein identifications, as well as the frequency of identification of proteins, varied between protocol groups. Protocol (f) demonstrated the highest number of proteins detected across all frequency categories. Most notably, it detected the greatest number of proteins with high frequency (>75%). Note: PSM, peptide–spectrum matches.

Proteins identified in this study were categorized into four groups based on their frequency of detection across all runs in each protocol (Supplemental Table S3): high (present in >75% of runs), medium (present in 50–74% of runs), low (present in 25–49% of runs), and rare (present in <25% of runs) (Figure 5 and Table 3). This classification provides critical insights into the consistency and reproducibility of protein identification across different protocols and experimental conditions. Among the identified proteins, 181 exhibited a high frequency of detection across all runs, with 73 of these proteins consistently identified in every run across all six protocols.

Such analyses are essential because identifying high-frequency proteins ensures confidence in their detection and establishes their potential as reliable candidates for further study. High-frequency proteins, especially those consistently detected across all runs, are more likely to represent core components of the tear proteome or proteins resistant to experimental variability. Notably, protocol (f) provided the largest number of high-frequency protein identification at t = 244, followed by (e) (t = 242), (d) (t = 220), (c) (t = 218), (b) (t = 213), and (a) (t = 174) (Figure 5 and Table 3). The superior performance of protocol (f) in identifying proteins with high frequency highlights its potential for achieving broader proteome coverage and greater reproducibility. By enabling the detection of proteins across a wide range of abundance levels, protocol (f) stands out as an optimal protocol for comprehensive proteomics analyses.

### 3.4. Protocol Variance

**Figure 6:**
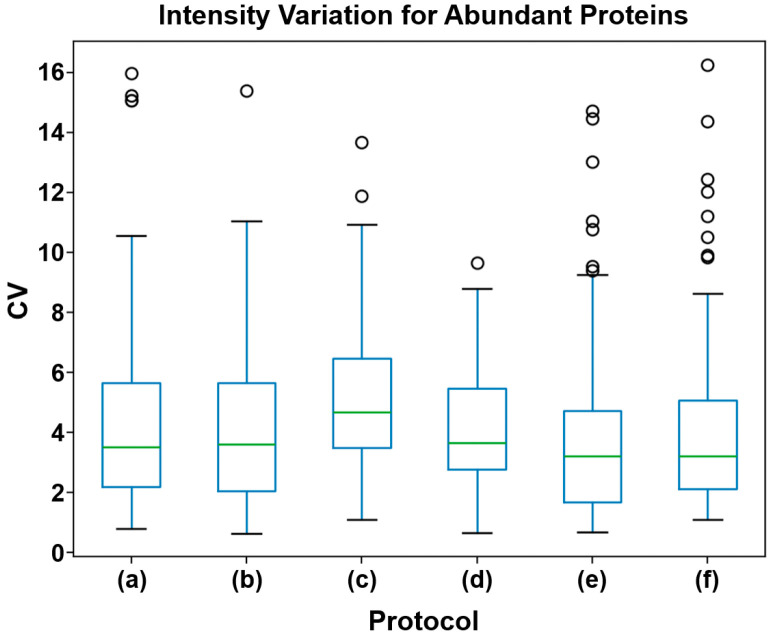
Comparison of CV% for high abundance proteins between protocols. Protocol (c) demonstrated the highest run-to-run variance, significantly higher than those observed for protocols (b), (d), (e), and (f). No other significant differences in variance were observed.

Understanding the variance between protocols is crucial to assessing the reproducibility and consistency of protein identification across multiple runs, which directly impacts reliability. Protocol (c), observed to have the highest average run-to-run variance at 5.20 ± 2.52, exhibited significantly higher variance when compared to protocols (b) (4.06 ± 2.64, *p* < 0.001), (d) (4.19 ± 2.12, *p* < 0.001) and (f) (4.36 ± 3.26, *p* = 0.02) (Figure 6 and Table 4). No significant difference in variance was observed between the other protocols. Protocol (e) had the lowest average variance at 3.96 ± 3.15 (Supplemental Table S4).

### 3.5. Protocol Selection

Selecting the optimal protocol requires balancing reproducibility with the ability to detect a wide range of proteins, including low-abundance and unique proteins. Despite not having the lowest variance, protocol (f) (with a total dilution volume of 50 µL and a 20:1 protein-to-trypsin ratio) emerged as the optimal choice for in-solution digestion due to several key advantages: (i) it identified the highest number of proteins and peptides, (ii) detected the greatest number of unique protein groups, and (iii) achieved the highest frequency of protein identification across all runs. Although protocol (f) did not exhibit the lowest run-to-run variance, its variance was not significantly higher than that of other protocols. Given its overall performance, protocol (f) provides the most comprehensive protein identification and is well-suited for further analyses. The reliability and effectiveness of protocol (f) make it the preferred option for tear proteomics.

### 3.6. Comparison of Pooled and Individual Samples

**Figure 7:**
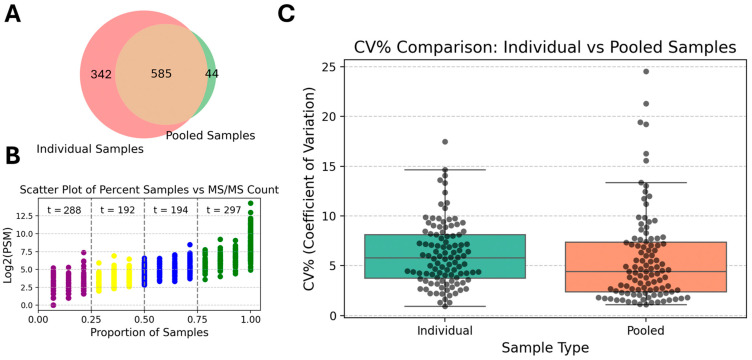
Comparison of proteins identified in individual small-volume tear fluid samples versus pooled samples run using protocol (f). (**A**) Individual sample runs identified 342 more proteins that were not detected in the pooled samples processed using the same processing protocol. (**B**) Across the individual sample runs 297 proteins were identified with high frequency (>75%), which is at the same level as the number of high-frequency proteins identified in the pooled sample. (**C**) The run-to-run variance was similar between the pooled and individual sample processing runs.

To evaluate the performance of protocol (f) in real tear proteomics studies, individual tear samples were processed using this optimized protocol and compared to the results from processing pooled tear fluid samples. A total of 971 unique proteins were identified in the individual samples (Supplemental Table S5). On average, 525 ± 123 proteins were identified across the fourteen samples; this was significantly greater than the 418 ± 95 identified on average when processing pooled samples using the same protocol (*p* = 0.013) (Figure 7A). In the individual sample runs, 297 proteins were identified with a high frequency (>75%), which is comparable to the number of high-frequency proteins found in the pooled sample (Figure 7B). Similar run-to-run variance was observed in both the pooled and individual sample runs. For many of the proteins consistently identified across both conditions, the individual samples did not exhibit significantly greater variation in run-to-run intensity than observed in the pooled samples (*p* = 0.302). This finding highlights that protocol (f) is a reliable and robust protocol for tear proteomics. Moreover, this protocol does not introduce significantly higher sample-to-sample variance relative to pooled samples (Figure 7C). Consequently, protocol (f) represents the optimized choice for tear proteomics and biomarker discovery.

### 3.7. Gene Ontology (GO) Enrichment Analysis and Tear Protein Classification

**Figure 8:**
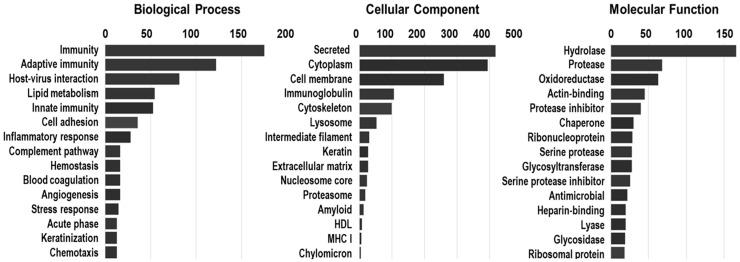
Gene Ontology (GO) enrichment analysis of tear fluid proteins showing their involvement in biological processes, cellular components, and molecular function.

GO enrichment analysis was conducted to categorize the proteins identified in tear fluid samples based on their biological processes, cellular components, and molecular functions (Figure 8). The biological process analysis revealed key functional roles of tear fluid proteins in innate and adaptive immunity, tissue homeostasis, and inflammation regulation. These findings underscore the importance of tear fluid in pathogen defense and immune response regulation [22]. Other processes, such as lipid metabolism, cell adhesion, and inflammatory response, underscore its involvement in wound healing and tissue maintenance. Cellular component analysis linked many proteins to secreted elements, cytoplasm, cell membranes, and immunoglobulin complexes, reinforcing tear fluid’s role in immune defense and cellular signaling. Molecular function analysis showed a predominance of hydrolase and protease activities, essential for enzymatic degradation and tissue remodeling, along with oxidoreductase, actin-binding, and protease inhibitor functions, reflecting tear fluid’s role in oxidative stress regulation and structural support [23]. Notably, lactotransferrin (LTF), lysozyme (LYZ), and prolactin-inducible protein (PIP), along with immunoglobulins, were highly expressed, showcasing tear fluid’s antimicrobial and anti-inflammatory proteins [24,25,26]. Albumin, a blood protein, indicates that tear fluid partly derives from blood filtrate, suggesting it may contain systemic disease biomarkers [5].

## 4. Discussion

Tear fluid is a rich source of protein biomarkers for both ocular and system diseases. Its non-invasive nature and ease of point-of-care collection make tear biomarkers a novel and promising approach for disease screening, diagnosis, and monitoring. As LC-MS/MS methods have improved, studies on tear fluid have explored its potential for diagnosing ocular conditions such as diabetic retinopathy, macular degeneration, and dry eye disease [2,6,8,9,12]. Furthermore, the eye’s connection to other body systems—such as tear fluid’s nature as an ultrafiltrate of blood and the link between the eye and the optic nerve—has made it an intriguing target for biomarker discovery in a wide range of systemic diseases, from breast cancer to Parkinson’s disease [2,9,12]. For many conditions, tear fluid holds significant potential for use in the development of cost-effective point-of-care tests as it can be collected non-invasively and with minimal training [11].

The development of rapid point-of-care tests could greatly improve access to care and patient outcomes. Diabetic retinopathy, for example, is a disease for which tear fluid biomarkers have been investigated, and approximately 90% of vision loss cases due to DR could have been prevented with a timely diagnosis [8,9,24]. Despite tear fluid’s potential for such testing, relatively few devices have been commercialized, primarily due to limited discovery and validation of tear fluid biomarkers. Therefore, developing improved methods for identifying and validating novel biomarkers could have a considerable long-term impact on diagnosis and care for a wide range of conditions.

LC-MS/MS-based proteomics is a powerful tool for identifying tear fluid biomarkers. However, the method of tear sample collection can significantly affect the results of shotgun proteomics analysis. Most LC-MS/MS-based proteomics analyses of tear fluid have been optimized for Schirmer strips [7,10,15,27,28,29,30,31,32,33,34,35,36,37,38,39,40,41,42,43,44,45,46,47,48,49,50,51,52,53,54,55,56], with a limited focus on optimizing methods for glass microcapillary tubes [1,23,31,57,58,59,60,61,62,63,64,65,66]. Existing methods for capillary tubes typically require larger sample volumes (5–10 µL), which can trigger significant reflex tearing, altering the sample composition and potentially reducing test reliability. Many methods use pooled tear fluid samples, which result in the loss of information on individual differences in tear fluid composition [1,8,31,57,58,59,60,62,63,64,66,67]. On average, across LC-MS/MS-based proteomics studies of tear fluid, Schirmer strips identify a larger number of proteins compared to studies employing glass capillary tubes—approximately 502 unique protein identifications on average for Schirmer strip studies compared to 140 for capillary studies [12]. These tubes, which mimic microfluidic processes used in point-of-care devices [68] offer several advantages over Schirmer strips [11,19]. Despite the potential benefits, the disparity in protein identification between capillary and Schirmer analysis methods significantly limits the utility of capillary collection for shot-gun discovery experiments. To date, more than 3000 unique proteins have been identified in tear fluid; as such, the low levels of protein identification in current methods have a high likelihood of missing potential biomarkers. For shotgun proteomics analysis, the optimization of protocols to detect a high number of unique proteins reliably greatly improves the chances of identifying novel markers of disease. To gain a comprehensive understanding of the tear fluid proteome, it is essential to optimize collection protocols for smaller-volume glass capillary tubes to enable single-collection analysis of tear fluid samples while minimizing cellular contamination and reflex tearing.

In this study, we developed an improved method for in-solution digestion of individual tear fluid samples collected using small-volume glass capillaries through the systematic optimization of digestion protocols. Protocol (f) was identified as the optimal method because it identified the highest number of proteins per run on average while maintaining low run-to-run variance. Using this method, we detected 1065 unique proteins in tear fluid across 30 pooled sample runs and 14 individual HC sample runs. We detected an average of 413 ± 116 proteins in the analysis of individual healthy control samples from a total volume of only 1 µL. This method significantly increases the number of unique proteins identified in capillary samples compared to the literature while also reducing the sample volume required for analysis. As tear fluid samples are known to be dynamic in composition at large volumes due to reflex tearing and the basal tear layer has an average volume of 3–10 μL, the application of this method reduces the impact of reflex tearing on sample analysis. This method additionally demonstrated high run-to-run reproducibility, and is efficient, requiring only 120 min of processing time without the need for fractionation.

This study further demonstrates the feasibility of “single-tear” proteomic analysis using LC-MS/MS for the identification of tear fluid biomarkers. In this study, we observed a higher number of protein identifications, with a similar run-to-run variance, between pooled samples and individual samples processed using identical protocols. The increased number of protein identifications observed in individual samples can be attributed to the varying dynamic ranges and differing protein concentrations across samples [69,70]. Processing low-volume individual tear fluid samples should be preferred in exploratory research, as it more reliably detects low-abundance proteins and preserves individual variation between subjects. Additionally, subjects with diseases relevant to tear fluid biomarker discovery, including dry eye disease and neurological conditions such as Parkinson’s disease, often experience reduced tear secretion due to disease pathology or medications used for treatment [4,25]. As a result, collecting sufficient sample volumes in many disease populations is often impossible or presents a significant barrier. Therefore, methods that optimize the reduction in initial sample volume can help expand the potential subject pool for patients with these diseases for exploratory research and improve the applicability of this work for future clinical translation.

Despite this improvement in analysis, the number of proteins detected remains lower compared to Schirmer strip methods, which have reported detecting as many as 1543 proteins in a single study [22]. The higher protein yield from Schirmer strips may be attributed to greater sample volumes and potential cellular contamination.

A limitation of this study is the lack of a direct comparison between capillary samples processed using this method and Schirmer strips, as no data were collected to compare the identified proteins between these methods. However, this improved capillary method will be valuable for making more representative comparisons between capillary and Schirmer collections in a future study. Additionally, since this study did not compare healthy control samples with those collected from disease groups, the full potential of this process for identifying novel biomarkers remains to be explored. Another limitation is that all samples were processed by a single operator at one site using a single instrument, so no information is available on the impact that processing by different technicians or at different sites may have on protein identification and variance. While this study does not explore potential differences in proteomic profiles in tear fluid based on gender, age, or ethnicity, it is expected that these methods can be applied to all capillary tear fluid samples regardless of these factors.

This approach expands the scope of proteomics studies by aligning sample analysis with collection methods suitable for diagnostic devices. Given the significant variations in tear fluid proteomics based on collection methods, it is crucial to establish reliable techniques for analyzing tear fluid across different sampling approaches. This will provide a more comprehensive understanding of the tear fluid proteome. Additionally, optimizing capillary processing techniques will improve comparisons between Schirmer and capillary sampling methods and enhance understanding of how sampling impacts analysis. This is essential for translating early discovery work into commercial assays and point-of-care diagnostics.

## Figures and Tables

**Table 1:** Demographics and ocular history of subjects included in pooled sample and individual samples.

Group	Pooled	Individual
Demographics		
Age (years)	63.36 ± 18.7 (20–92)	53 ± 23.2 (23–89)
Gender (male) *	19/35	2/14
Ocular Health		
Diabetic Retinopathy	2/35	
Cataract	6/35	
Dry Eye	1/35	
Glaucoma	5/35	
Age-Related Macular Degeneration	7/35	
Collection Time		
Morning (9:30 a.m.–12:00 p.m.)	21/35	11/14
Afternoon (12:00 p.m.–5:00 p.m.)	14/35	3/14

* Gender is represented as proportion of male subjects over the total.

**Table 2:** Descriptive statistics comparing protein and peptide numbers between protocols. Note: STD, standard deviation.

Protocol	Peptides		Proteins	
	Average	STD	Average	STD
(a)	835	181	293	35
(b)	1088	381	352	28
(c)	1095	400	365	76
(d)	1085	253	360	57
(e)	1017	160	363	14
(f)	1270	314	418	95

**Table 3:** The number of proteins identified by each protocol is separated by the frequency of identification.

Unique Proteins Identified	Protocol					
	(a)	(b)	(c)	(d)	(e)	(f)
High Abundance (>75%)	174	213	218	220	242	244
Medium Abundance (50–75%)	65	70	73	73	74	90
Low Abundance (25–49%)	72	88	89	85	80	133
Rare (<25%)	95	137	170	138	138	162
Total	406	508	550	516	534	629

**Table 4:** CV% for selected highly abundant tear fluid proteins by protocol with descriptive statistics.

Protein Name	(a)	(b)	(c)	(d)	(e)	(f)
Lactotransferrin	1.09	1.83	1.96	1.68	1.04	1.67
Lysozyme C	2.08	1.91	2.55	2.79	1.95	2.29
Basement membrane-specific heparan sulfate proteoglycan core protein	2.05	1.35	3.56	2.30	1.45	2.25
Albumin	1.84	2.06	3.25	2.28	1.44	2.47
Mammaglobin-B	2.51	6.04	5.44	7.53	3.68	3.22
Cystatin-S	4.37	5.36	6.37	3.61	3.64	4.91
Proline-rich protein 4	3.25	4.55	4.31	5.81	1.39	2.70
Cystatin-C	5.08	5.29	8.15	5.04	7.96	6.04
Average	4.56	4.06	5.20	4.19	3.96	4.36
STD	3.47	2.64	2.52	2.12	3.15	3.26

## Data Availability

The mass spectrometry proteomics data have been deposited to the ProteomeXchange Consortium via the PRIDE [26] partner repository with the dataset identifier PXD060101.

## Supplementary Materials

The following supporting information can be downloaded at: https://www.mdpi.com/article/10.3390/biomedicines13020386/s1, Supplemental Table S1: The 1022 Tear Proteins Identified in This Study; Supplemental Table S2: Tear Proteins Identified in Pooled Tear fluid Experiments to Optimize LC-MS/MS Digestion Protocol; Supplemental Table S3: The Frequency of Detection Across Experimental Groups for 1022 Tear Proteins Identified in This Study; Supplemental Table S4: Intra-protocol CV of proteins identified across all pooled sample runs; Supplemental Table S5: Tear Proteins Identified Individual Samples Processed using Protcol F.
